# Supplementary material for: Transposon end recognition and pairing by I-F3 CRISPR-associated transposase
Source: bioRxiv. 2026 Jul 10:2026.07.09.737429. Preprint. [Version 1] doi: 10.64898/2026.07.09.737429 (PMC13370975; doi:10.64898/2026.07.09.737429)
Supplement: Supplement 1 [file NIHPP2026.07.09.737429v1-supplement-1.pdf]

bioRxiv preprint doi: https://doi.org/10.64898/2026.07.09.737429; this version posted July 10, 2026. The copyright holder for this preprint (which was not certified by peer review) is the author/funder, who has granted bioRxiv a license to display the preprint in perpetuity. It is made available under a CC-BY 4.0 International license.

Supplemental Figures

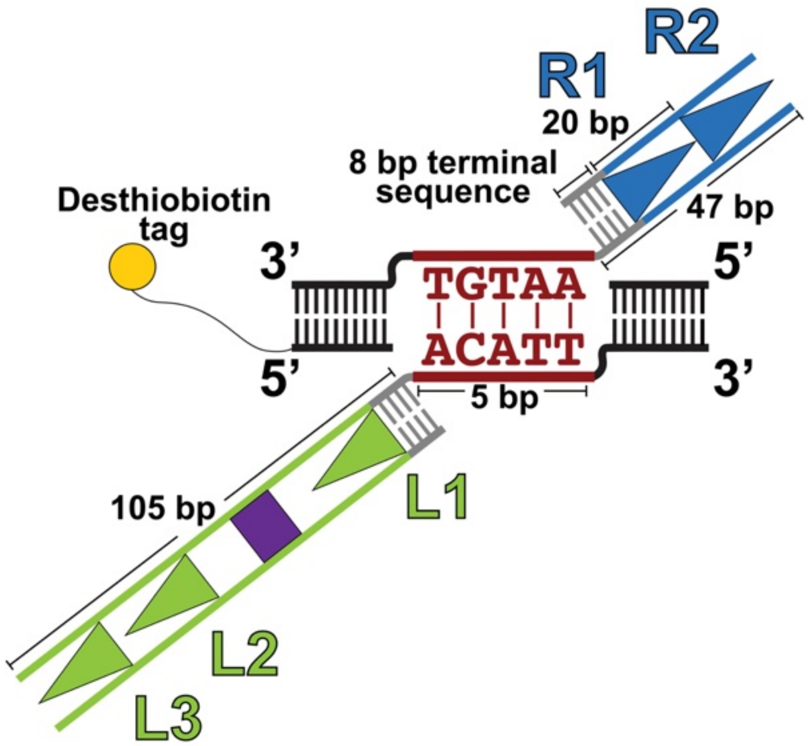

Supplemental Figure 1. Schematic of the designed asymmetric strand-transfer DNA substrate, representing the Shapiro intermediate product of the VchCAST strand-transfer reaction. The 105-bp segment of the transposon left end (green) contains three TnsB binding sites (L1–L3; each site is 20 nucleotides, indicated by triangles) and an IHF-binding site (purple). The 47-bp segment of the transposon right end (blue) includes two TnsB binding sites (R1–R2). Each end contains an 8-bp terminal sequence (gray), while the target DNA is shown in black. A desthiobiotin tag (yellow circle) is attached to the target DNA to facilitate affinity pulldown during sample reconstitution.

St. Jude - Confidential

bioRxiv preprint doi: https://doi.org/10.64898/2026.07.09.737429; this version posted July 10, 2026. The copyright holder for this preprint (which was not certified by peer review) is the author/funder, who has granted bioRxiv a license to display the preprint in perpetuity. It is made available under a CC-BY 4.0 International license.

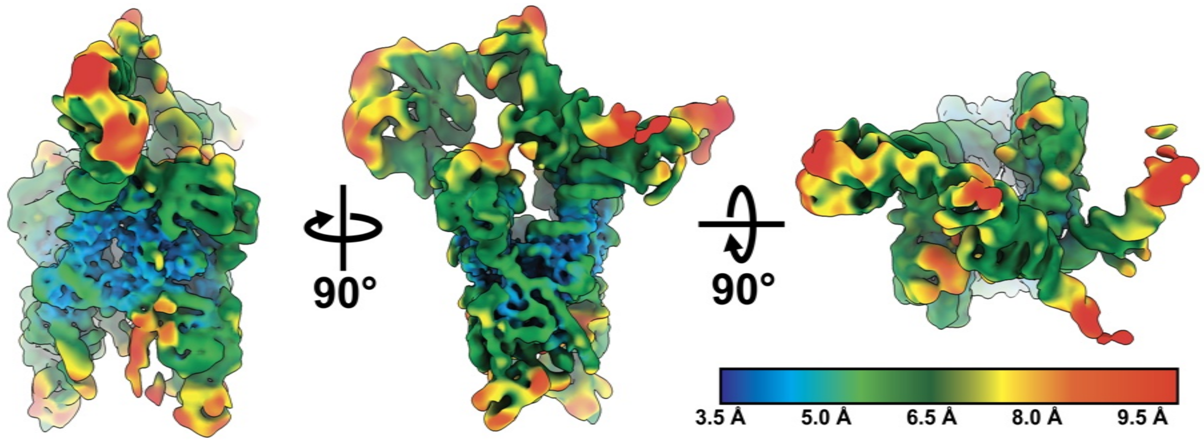

Supplemental Figure 2. Local resolution map of consensus map. Map voxels are colored according to local resolution estimates; color legend is on bottom right of figure. Three different views of the locally filtered consensus map are shown, labeled by rotations required to relate the different views. Map shown corresponds to the locally filtered consensus map, displayed at threshold = 0.0936. Local resolution was assessed using the local resolution estimation tool in CryoSPARC, applying a Fourier shell correlation (FSC) threshold of 0.5.

bioRxiv preprint doi: https://doi.org/10.64898/2026.07.09.737429; this version posted July 10, 2026. The copyright holder for this preprint (which was not certified by peer review) is the author/funder, who has granted bioRxiv a license to display the preprint in perpetuity. It is made available under a CC-BY 4.0 International license.

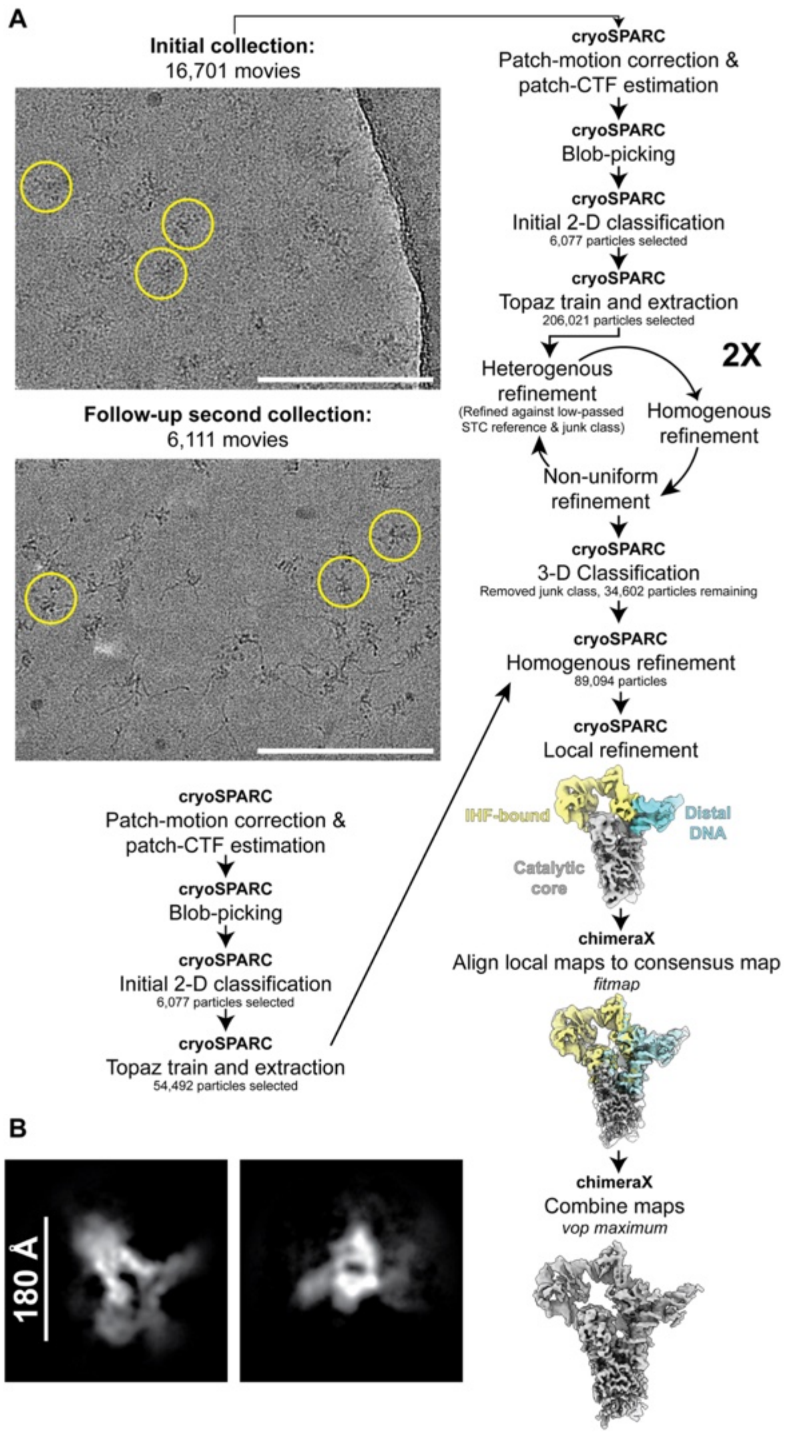

Supplemental Figure 3. Image processing workflow to produce composite map. Representative micrographs are shown from each data collection session, with yellow circles highlighting examples of particles. White scale bar represents 150 nm. Image processing workflow from pre-processing (top) to final composite map reconstruction (at bottom). The output of three separate local refinement jobs focused on different regions: IHF-bound region, catalytic core, and distal end DNA. Locally refined maps are shown below in different colors: yellow, grey, and light blue respectively. **B.** Reference-free 2D classes from collected dataset. Scale bar represents 180 Å.

bioRxiv preprint doi: https://doi.org/10.64898/2026.07.09.737429; this version posted July 10, 2026. The copyright holder for this preprint (which was not certified by peer review) is the author/funder, who has granted bioRxiv a license to display the preprint in perpetuity. It is made available under a CC-BY 4.0 International license.

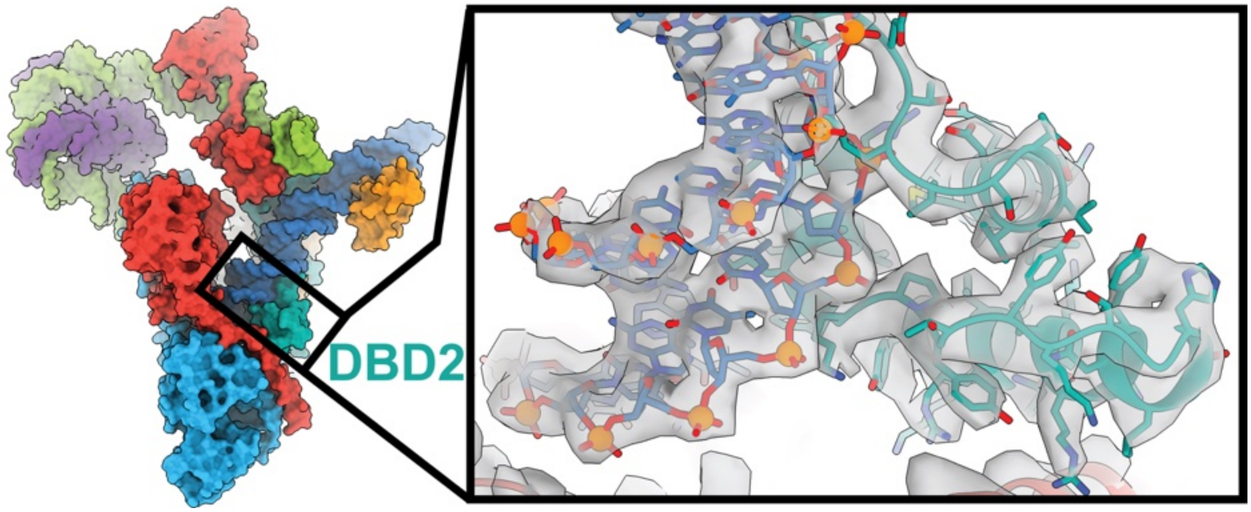

Supplemental Figure 4. Highest resolution structural features observed in the catalytic core. Left: structure overview provided as a reference for the reader, boxed region indicates location of close-up, along with a label for the domain being visualized. Right: Close-up view of the atomic model, shown in sticks, of DBD2 (teal) associated with RE1 (blue) superimposed with the locally-refined map of the catalytic core (gray surface). Atoms are colored according to element: oxygen is orange, nitrogen is blue, phosphates are orange. The map threshold (threshold = 0.622) was chosen to highlight the separation between DNA bases.

bioRxiv preprint doi: https://doi.org/10.64898/2026.07.09.737429; this version posted July 10, 2026. The copyright holder for this preprint (which was not certified by peer review) is the author/funder, who has granted bioRxiv a license to display the preprint in perpetuity. It is made available under a CC-BY 4.0 International license.

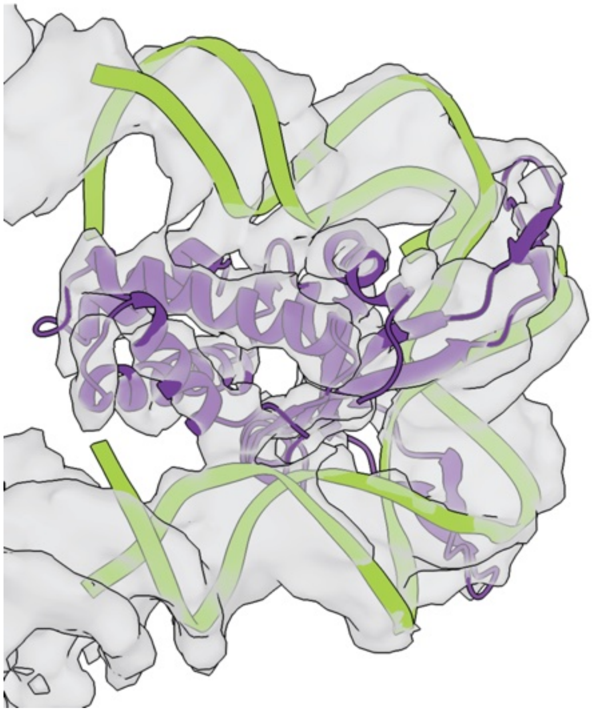

Supplemental Figure 5. The crystal structure of previously determined IHF structure docked into the composite map. The co-crystal structure of *E. coli* IHF-DNA (PDB 1IHF) is rigid body docked into the VchCAST locally-refined IHF-bound region composite map (transparent gray surface, threshold = 0.44), showcasing the 180° DNA bend generated by the host factor. Protein is shown in purple cartoon, DNA backbone displayed in green ribbon.

bioRxiv preprint doi: https://doi.org/10.64898/2026.07.09.737429; this version posted July 10, 2026. The copyright holder for this preprint (which was not certified by peer review) is the author/funder, who has granted bioRxiv a license to display the preprint in perpetuity. It is made available under a CC-BY 4.0 International license.

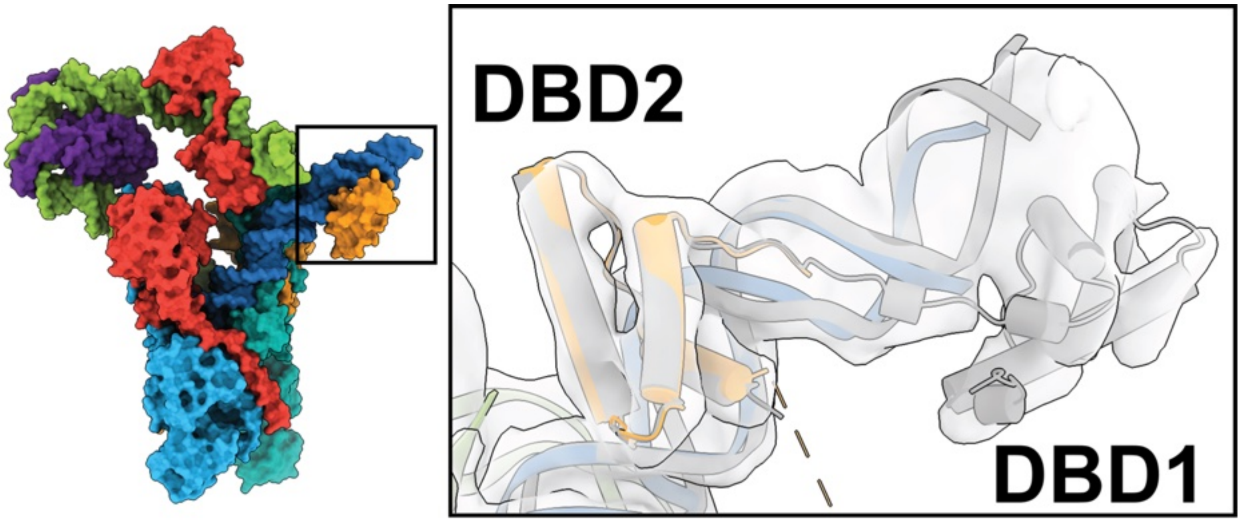

Supplemental Figure 6. Expected location of DBD1 on RE2. Left: structure overview shown as a reference for the reader, boxed region indicates location of the close-up. Right: Locally filtered consensus cryo-EM map shown in transparent gray (threshold = 0.087). Although the DBD1 domain of TnsB-RE2 was not built due to weak density, superposition with the same region of TnsB-LE1 (cartoon representative, colored gray, DBD1 and DBD2 labeled) shows its expected location.

bioRxiv preprint doi: https://doi.org/10.64898/2026.07.09.737429; this version posted July 10, 2026. The copyright holder for this preprint (which was not certified by peer review) is the author/funder, who has granted bioRxiv a license to display the preprint in perpetuity. It is made available under a CC-BY 4.0 International license.

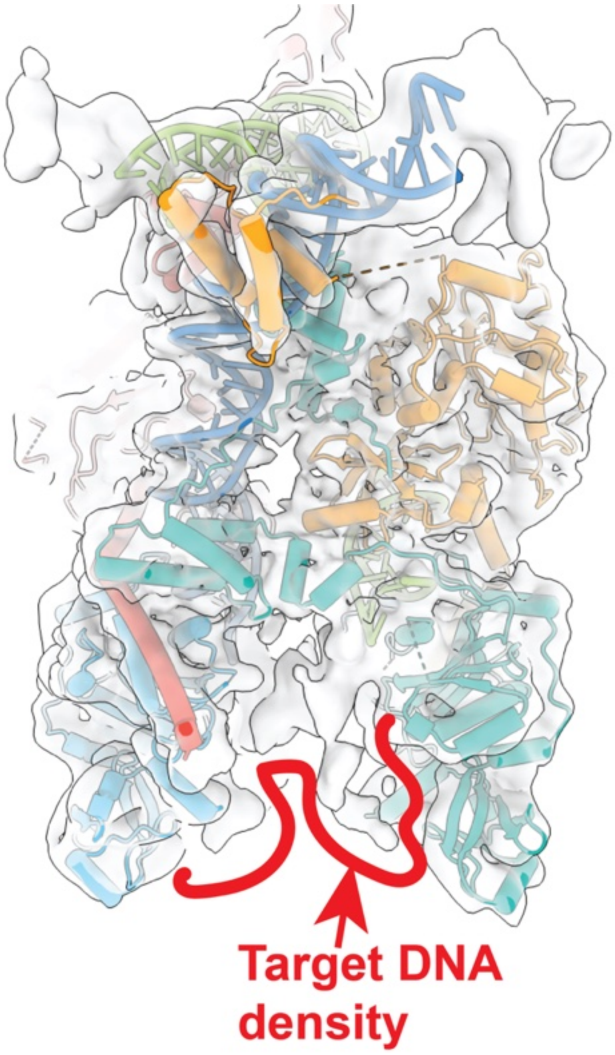

Supplemental Figure 7. Cryo-EM map exhibits weak target DNA density. Locally filtered consensus map displayed at 0.082 threshold, in the same viewing direction as shown in Figure 2A. Map shown in transparent gray surface. Atomic model, shown in cartoon is docked into the map, colors are the same as throughout the manuscript. Target DNA density is partially outlined by the red line to facilitate visualization.

bioRxiv preprint doi: https://doi.org/10.64898/2026.07.09.737429; this version posted July 10, 2026. The copyright holder for this preprint (which was not certified by peer review) is the author/funder, who has granted bioRxiv a license to display the preprint in perpetuity. It is made available under a CC-BY 4.0 International license.

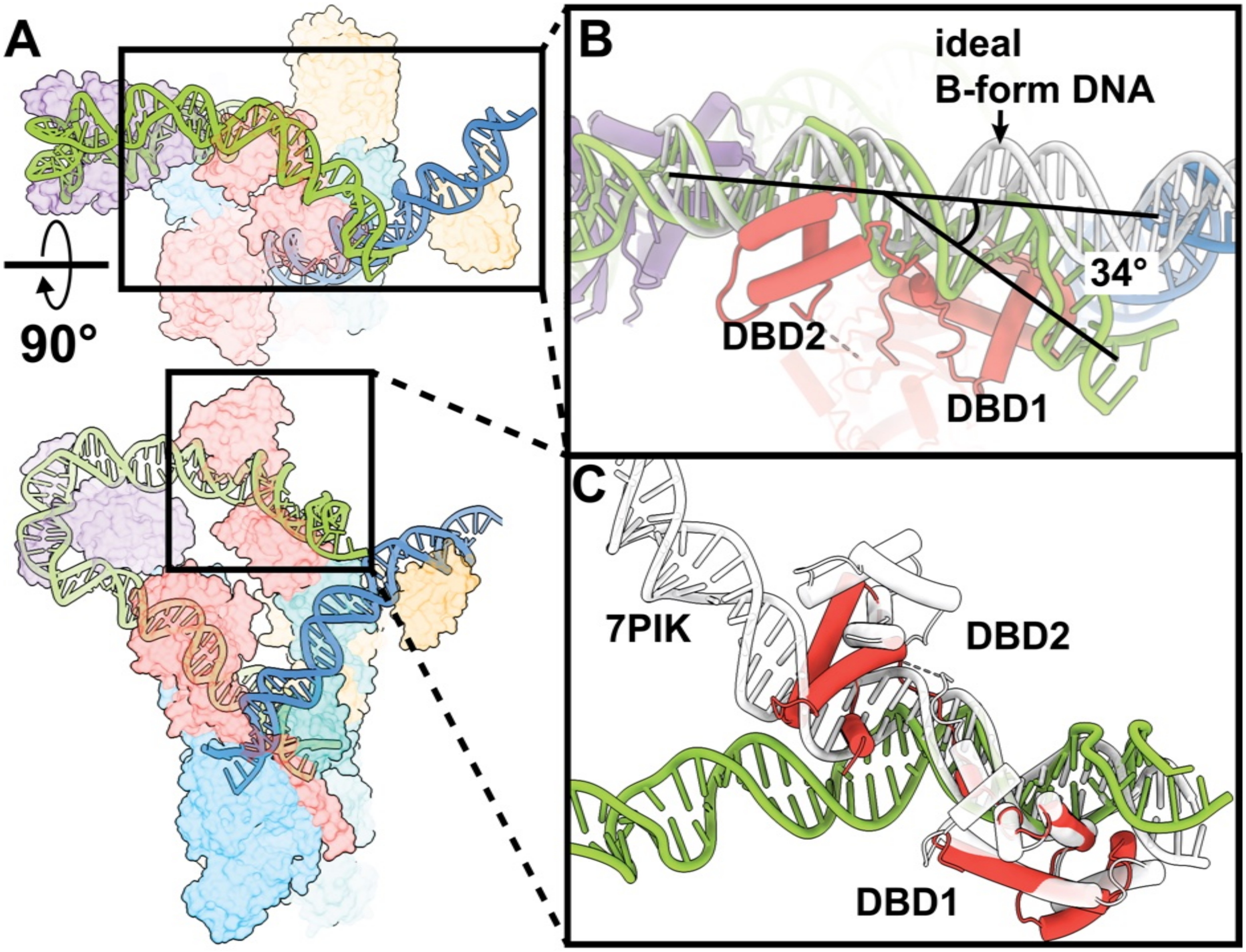

Supplemental Figure 8. Local DNA distortions observed and compared to previously determined structures. A. Transparent surface representation of the atomic structure shown in two orthogonal views. Color scheme follows that of figure 1. DNA structure is shown in cartoon to facilitate visualization. Callouts (boxes) indicate the portions of the structure shown in greater detail in panels B and C. Rotation axis/angle indicates the rotation in view going from the bottom panel to the top panel. **B.** Cartoon representation of the atomic structure superimposed onto a model of ideal B-form DNA (white, ribbon). The left end is colored light green, and DNA-binding domains (DBD1 and DBD2) are labeled. The bending angle between ideal B-form DNA and the left end is shown (black lines) on top of the displayed structures. **C.** Previously determined Tn7 TnsB structure (PDB: 7PIK, white cartoon) is superimposed onto DBD1 of TnsB-LE2 (colored cartoon). DBD1 and DBD2 are labeled.

St. Jude - Confidential

bioRxiv preprint doi: https://doi.org/10.64898/2026.07.09.737429; this version posted July 10, 2026. The copyright holder for this preprint (which was not certified by peer review) is the author/funder, who has granted bioRxiv a license to display the preprint in perpetuity. It is made available under a CC-BY 4.0 International license.

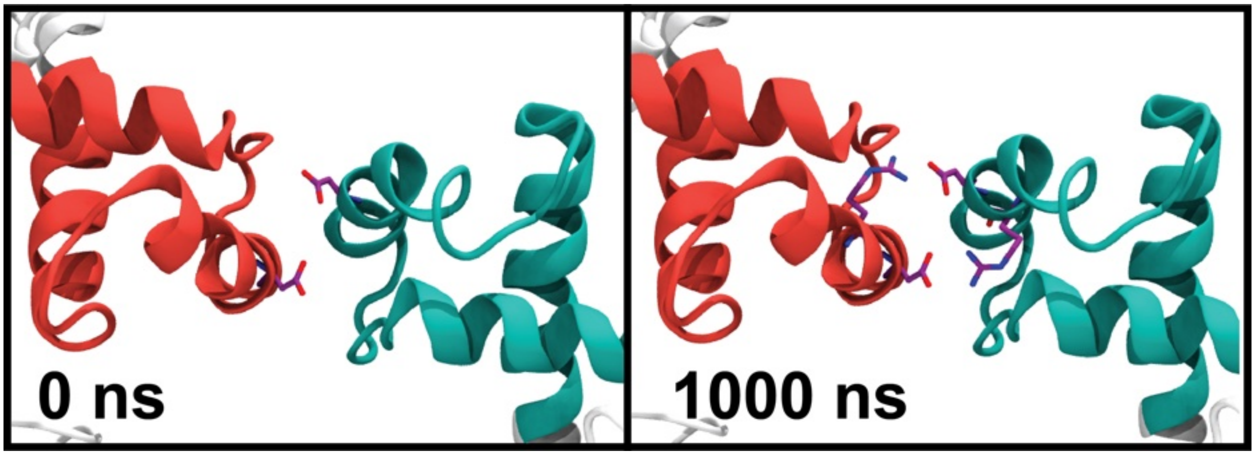

Supplemental Figure 9. Molecular dynamics (MD) simulation snapshots of the V78D mutant showing compensatory interaction of V78D residues with neighboring Arginine residues (R38) at the TnsB-LE2–TnsB-RE1 interface, resulting in lack of separation. Timepoint in nanoseconds is indicated at the bottom left of each panel.

bioRxiv preprint doi: https://doi.org/10.64898/2026.07.09.737429; this version posted July 10, 2026. The copyright holder for this preprint (which was not certified by peer review) is the author/funder, who has granted bioRxiv a license to display the preprint in perpetuity. It is made available under a CC-BY 4.0 International license.

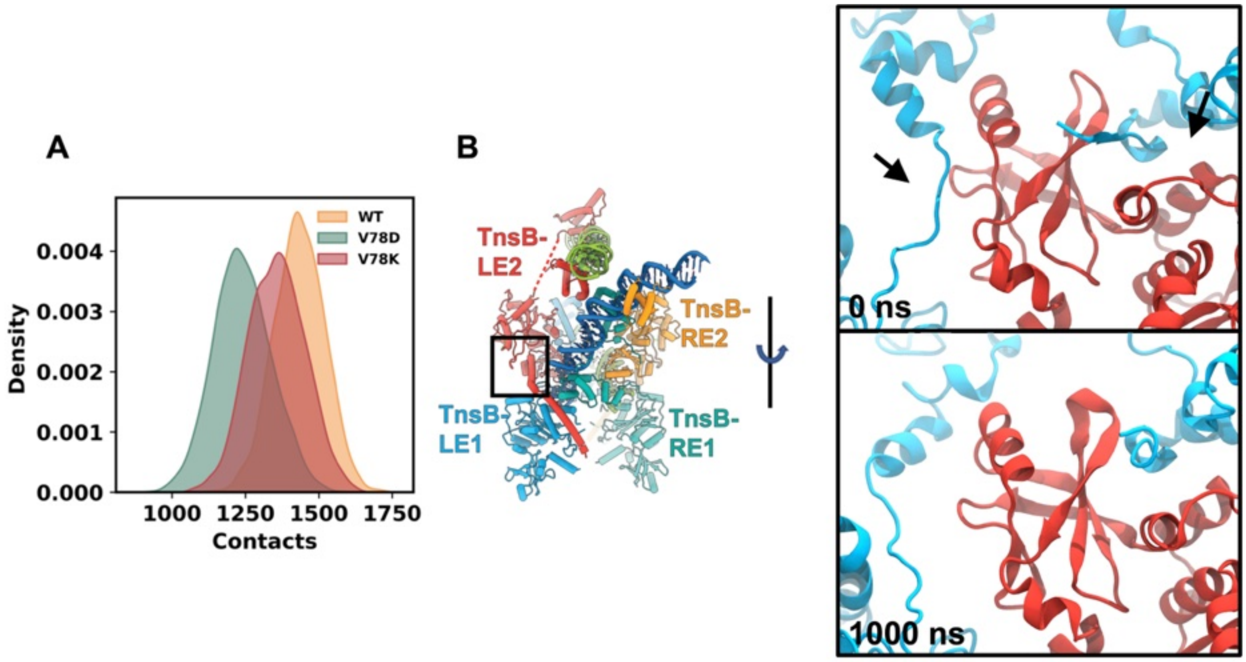

Figure S10: A. Density plots showing number of contacts between of LE2–LE1 inter-subunit from MD simulations of WT and mutant system. **B.** left: overview of interface being analyzed in panel A. right: MD snapshots of the V78D mutant, arrows highlight the regions at TnsB-LE2 — TnsB-RE1 interface that contributed to reduction in contacts.

bioRxiv preprint doi: https://doi.org/10.64898/2026.07.09.737429; this version posted July 10, 2026. The copyright holder for this preprint (which was not certified by peer review) is the author/funder, who has granted bioRxiv a license to display the preprint in perpetuity. It is made available under a CC-BY 4.0 International license.

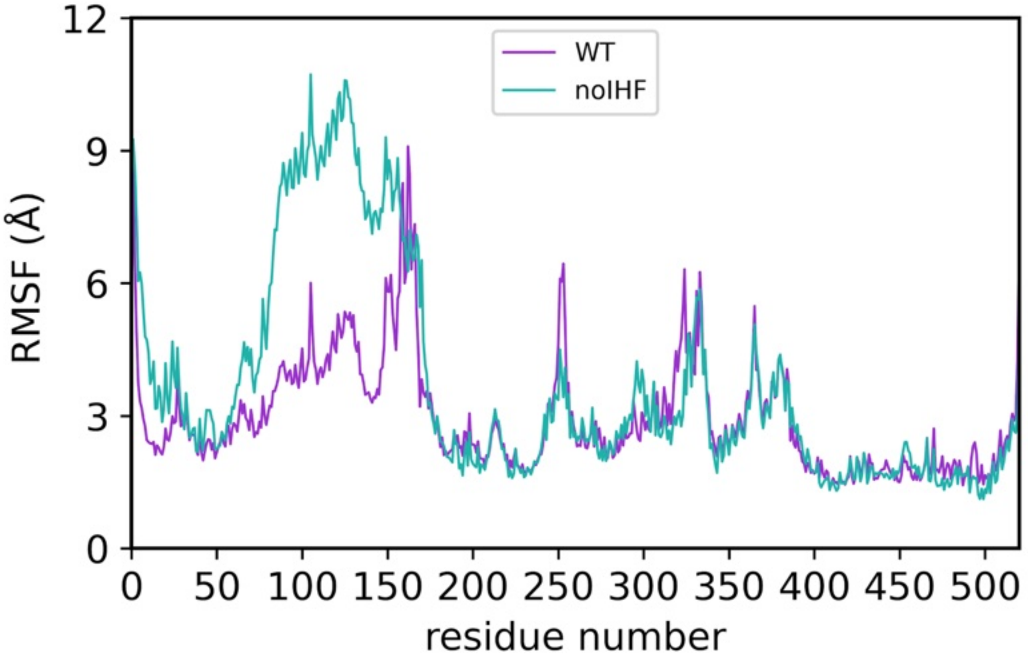

Supplemental Figure S11. RMSF on a per-residue basis for TnsB-LE2. Residue number plotted on the x-axis, root-mean-square-fluctuation (RMSF) on the y-axis. WT include the full assembly with IHF bound (pink), noIHF corresponds to the the assembly lacking IHF (green).

bioRxiv preprint doi: https://doi.org/10.64898/2026.07.09.737429; this version posted July 10, 2026. The copyright holder for this preprint (which was not certified by peer review) is the author/funder, who has granted bioRxiv a license to display the preprint in perpetuity. It is made available under a CC-BY 4.0 International license.

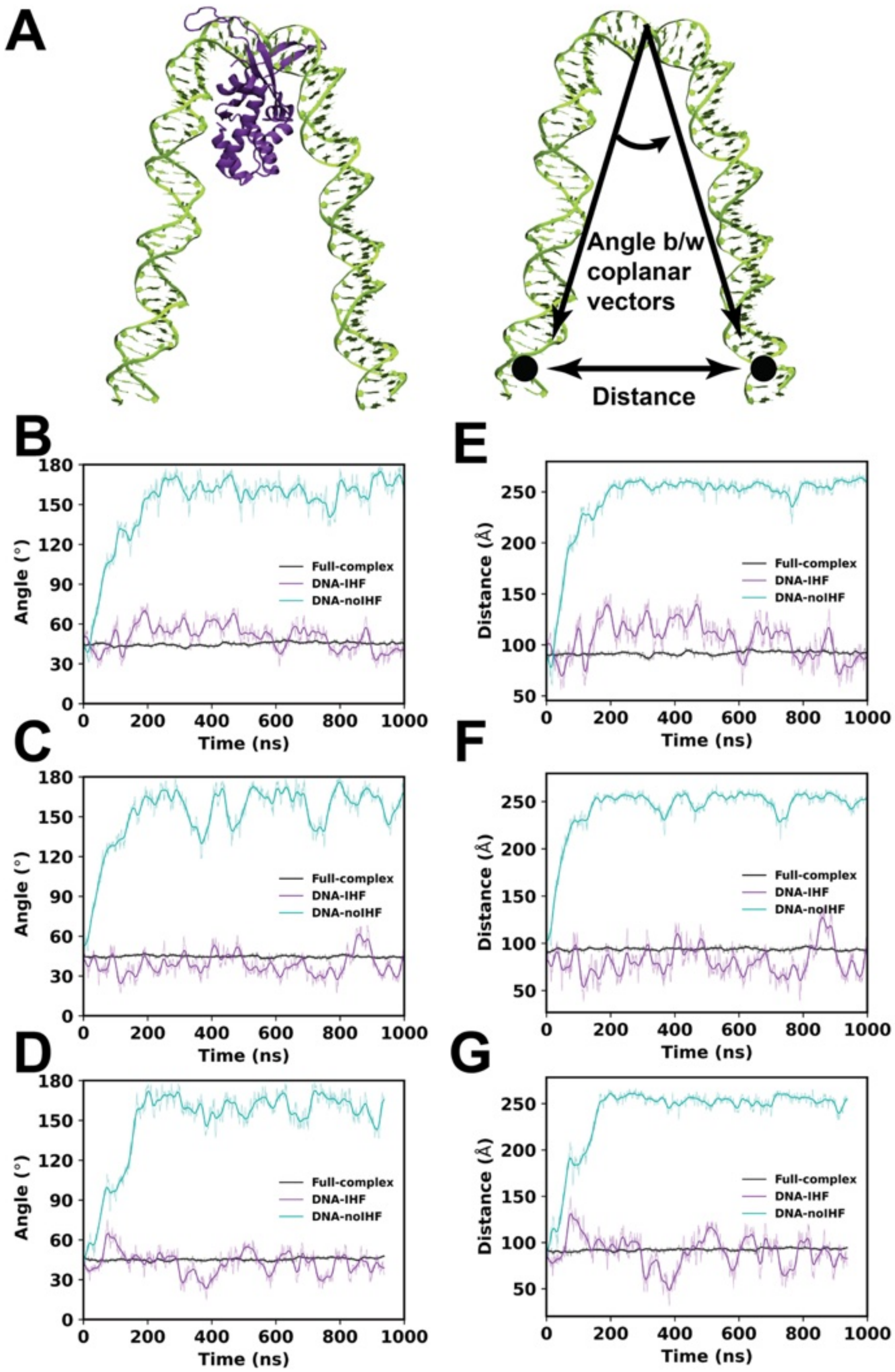

Supplemental Figure 12. IHF stabilizes the LE-DNA conformation. Atomic model of the LE-DNA in presence of IHF (left panel) and in absence of IHF (right panel). A schematic illustration showing two coplanar vectors defined along the DNA. The angle between the vectors (curved arrow) captures their relative orientation, while the linear distance between their base points (black circles) represents the end-to-end distance. **B–D,** Time evolution of the vector angle for three independent replicas of LE-DNA simulated in the presence and absence of IHF. **E–G,** Corresponding end-to-end distances for the same replicas, illustrating the effect of IHF binding on LE-DNA conformation.

bioRxiv preprint doi: https://doi.org/10.64898/2026.07.09.737429; this version posted July 10, 2026. The copyright holder for this preprint (which was not certified by peer review) is the author/funder, who has granted bioRxiv a license to display the preprint in perpetuity. It is made available under a CC-BY 4.0 International license.

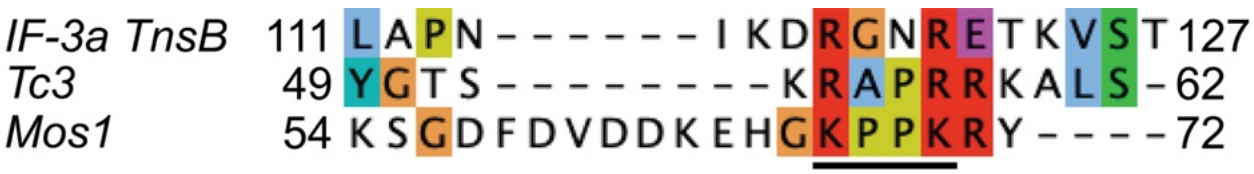

Supplemental Figure 13. ‘AT-hook’-like motif and DBD1-DBD2 linker sequence alignment. Sequence alignment of homologous segments from related transposons: Tc3 and Mos1. ‘AT-hook’-like motif is underlined. Residues are colored according to their biophysical properties and conservation, with the bounding residue numbers shown at the beginning and end of the alignment.

bioRxiv preprint doi: https://doi.org/10.64898/2026.07.09.737429; this version posted July 10, 2026. The copyright holder for this preprint (which was not certified by peer review) is the author/funder, who has granted bioRxiv a license to display the preprint in perpetuity. It is made available under a CC-BY 4.0 International license.

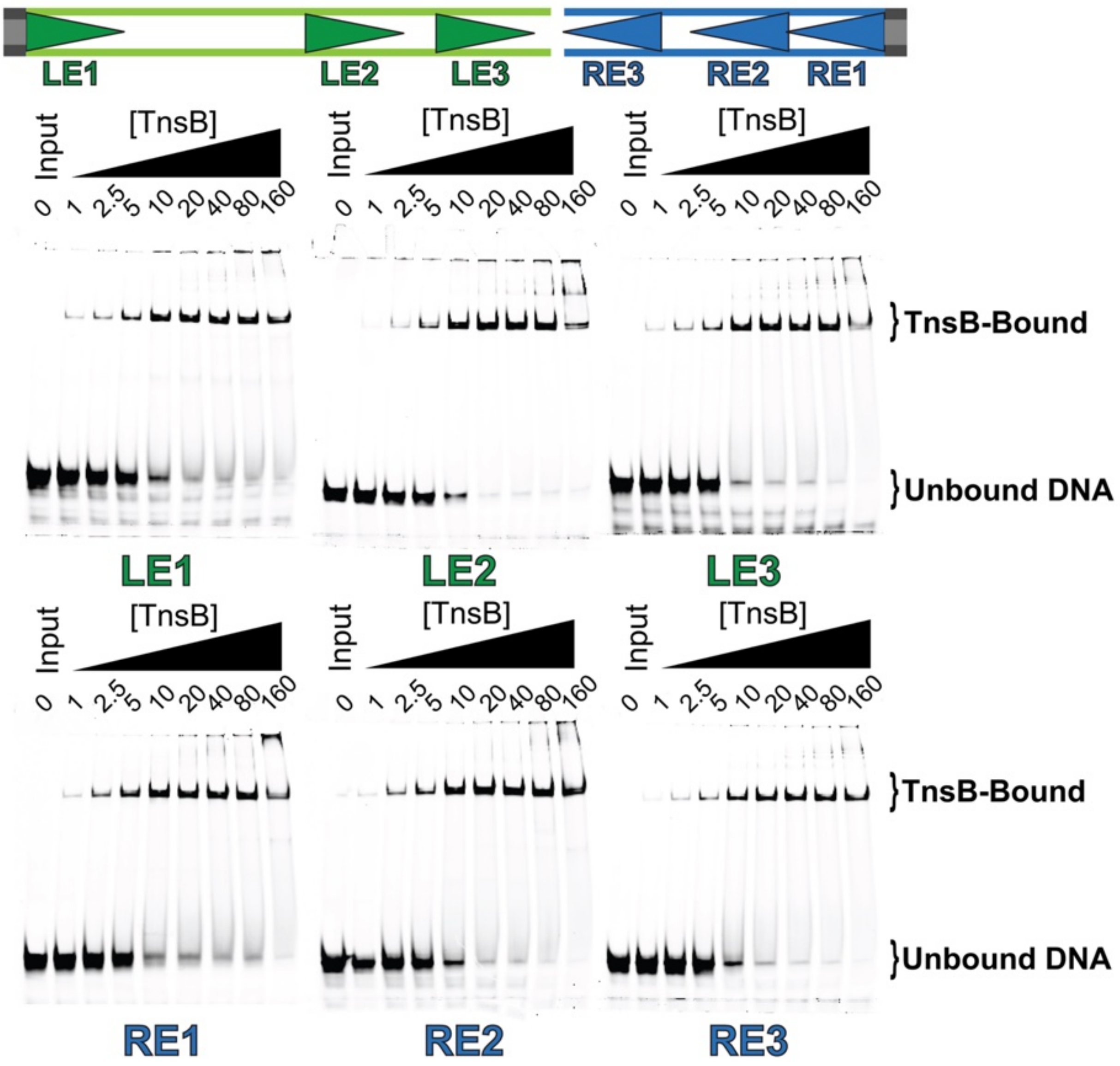

Supplemental Figure 14. Transposase binding affinities measured across all transposase binding sites by electrophoretic mobility shift assays (EMSA). EMSA quantification of tranposase binding site affinities with TnsB. Oligos representing each individual binding site within the transposon ends are annealed and reconstituted with range of TnsB concentrations. DNA is supplied in each lane at 5 nM. Bands under the unbound DNA region represent misannealed/free DNA.

bioRxiv preprint doi: https://doi.org/10.64898/2026.07.09.737429; this version posted July 10, 2026. The copyright holder for this preprint (which was not certified by peer review) is the author/funder, who has granted bioRxiv a license to display the preprint in perpetuity. It is made available under a CC-BY 4.0 International license.

Supplemental movie 1: Architecture of the VchCAST transposase complex with synapsed ends. Three-dimensional rotational views of the cryo-EM map (threshold = 8.97) are shown, both alone and alongside the atomic model. The map is colored as in Figure 1 to distinguish different components of the complex. Results from CryoSPARC 3DFlex heterogeneity analysis are also presented. Heterogeneity maps (threshold = 0.0352) are low-pass filtered to illustrate dynamics at 8 Å resolution.

St. Jude - Confidential

bioRxiv preprint doi: https://doi.org/10.64898/2026.07.09.737429; this version posted July 10, 2026. The copyright holder for this preprint (which was not certified by peer review) is the author/funder, who has granted bioRxiv a license to display the preprint in perpetuity. It is made available under a CC-BY 4.0 International license.

Oligonucleotides

Name	Description	Sequence	Used Figure(s)
LE-Target	Complex reconstitution substrate	ATATCAACTTATGGTTGTTTTGTGAGATATCAATATATGGTTGTTTTGTGGTTAAGTTGCTGATTATAAATAATTATTAAATATCACTTTATGGTTGCATCAACAACATTCCATCGTCTGCTCATCCATG	1, 2, S1-6, S8
LE-TargetFlank-RC	Complex reconstitution substrate	CATGGATGAGCAGACGATGG	1, 2, S1-6, S8
LE-HOST	Complex reconstitution substrate	CACAGCGGCTCTTTTTGATTTGTTGATGCAACCATAAAGTGATATTTAATAATTATTTATAATCAGCAACTTAACCACAAAACAACCATATATTGATATCTCACAAAACAACCATAAGTTGATAT	1, 2, S1-6, S8
DTB-LUEGO	Complex reconstitution substrate	/5deSBioTEG/GTGCCCTGGTCTGG	1, 2, S1-6, S8
NTS-StructuralBubble	Complex reconstitution substrate	TTCATCAAGCCATTGGACCGCCTTACACGACGCATTGGCATCATTCCTTTTGACAGCGCACGGCGTTAAAGTTGTTCTGCTTCATCAGCAGGATATCCTGCA	1, 2, S1-6, S8
TargetStrand-RE	Complex reconstitution substrate	AATTATCAATTTATGGGTGTAATTATCATTTTATGGTTGTATCAACAAATGTTGCAGGATATCCTGCTGATGAAGCAGAACAACTTTAACGCCGTGCGCTGTGAAAAGCAATGAAGCCAAAGCGTCCTGTAAGGCGGTCCAATGGCTTGATGAACCAGACCAGGGCAC	1, 2, S1-6, S8
RE-HOST	Complex reconstitution substrate	GTAAGCGCATAGATTCTCTGTGTTGATACAACCATAAAATGATAATTACACCCATAAATTGATAATT	1, 2, S1-6, S8
AlaSwap-F	Forward primer for V78A TnsB mutation	ACCACATCGTGCGGAGCTACAGC	1E
AspSwap-F	Forward primer for V78D TnsB mutation	ACCACATCGTGATGAGCTACAGC	1E
PhenylSwap-F	Forward primer for V78F TnsB mutation	ACCACATCGTTTTGAGCTACAGC	1E
LysSwap-F	Forward primer for V78K TnsB mutation	ACCACATCGTAAAGAGCTACAGC	1E

bioRxiv preprint doi: https://doi.org/10.64898/2026.07.09.737429; this version posted July 10, 2026. The copyright holder for this preprint (which was not certified by peer review) is the author/funder, who has granted bioRxiv a license to display the preprint in perpetuity. It is made available under a CC-BY 4.0 International license.

ResSwap-R	Reverse primer for V78 TnsB mutations	TCAATTGATTTGGCCACAATG	1E
CatMutant-TnsB-F	Forward primer for D308A TnsB mutation	ACTCGTAACTGCGAATGGTAAAGAGTTTTTG	1E
CatMutant-TnsB-R	Reverse primer for D308A TnsB mutation	AGGTCTGGGATGCCATAA	1E
